# Longitudinal Associations Between Cultural Engagement and Mental and Social Well-Being: A Fixed-Effects Analysis of the English Longitudinal Study of Ageing

**DOI:** 10.1093/geronb/gbaf074

**Published:** 2025-04-22

**Authors:** Saoirse Finn, Jessica K Bone, Daisy Fancourt, Katey Warran, Hei Wan Mak

**Affiliations:** Department of Behavioural Science & Health, Institute of Epidemiology & Health Care, University College London, London, UK; Department of Behavioural Science & Health, Institute of Epidemiology & Health Care, University College London, London, UK; Department of Behavioural Science & Health, Institute of Epidemiology & Health Care, University College London, London, UK; Department of Behavioural Science & Health, Institute of Epidemiology & Health Care, University College London, London, UK; Public Health, Arts, Theory, Sociology (PATHS) Research Group, School of Health in Social Science, University of Edinburgh, Edinburgh, UK; Department of Behavioural Science & Health, Institute of Epidemiology & Health Care, University College London, London, UK; (Social Sciences Section)

**Keywords:** Arts engagement, Generalized method of moments, Leisure, Loneliness, Mental health

## Abstract

**Objectives:**

Cultural engagement (e.g., going to museums, the theater, and concerts) has been evidenced to support older adults’ well-being. However, whether cultural engagement is associated with multiple well-being domains and whether associations vary by sociodemographics and health warrants further investigation.

**Methods:**

Using 14 years of data from the English Longitudinal Study of Ageing, we tested the longitudinal associations between cultural engagement and 7 well-being outcomes among 6,932–10,428 individuals aged 50–99 years. We used fixed-effects regression to explore the longitudinal associations between cultural engagement and the outcomes, generalized method of moments estimators to assess directionality, and interactions to test for moderation effects of sociodemographic and health conditions.

**Results:**

We found that increases in cultural engagement were associated with increases in life satisfaction, quality of life, happiness, and having a worthwhile life and decreases in depressive symptoms, anxiety, and loneliness. After assessing directionality, cultural engagement increases predicted decreases in depressive symptoms. Interactions suggest that older adults with a long-standing health condition, living without a partner, and who are female may experience greater well-being benefits from being culturally engaged.

**Discussion:**

Our findings underscore the potential of cultural engagement to enhance multiple well-being domains for older adults. This emphasizes the need to ensure equitable access to cultural engagement for all older adults, particularly those facing barriers to participation and those with poorer health, who may benefit the most from such initiatives.

The world’s population is aging. Worldwide, the number of people aged 65 years or older is estimated to rise from one in 10 in 2021 to one in six in 2050 (United Nations, 2023). In the U.K., approximately one in five people is aged 65 and above (Centre for Ageing Better, 2023). While people are living longer, they are not necessarily living happier. Aging poses challenges to an individual’s mental and social well-being. Life transitions synonymous with aging (including retirement, reduced social networks, poorer physical health, and decline in mobility) can heighten poorer mental and social well-being, including greater loneliness and depression as people age (Dahlberg et al., 2021; Zenebe et al., 2021). In addition to this, the aging population has led to an increased need for healthcare services treating older adults with complex and long-term healthcare needs. However, these healthcare systems are underfunded and overstretched, exacerbating the challenges older adults face (Gentry et al., 2023). Consequently, exploring alternative lifestyle-oriented public health approaches is crucial to support older adults’ mental and social well-being.

Cultural engagement (e.g., visits to cinemas, museums, and concerts) is one public health approach that could support mental and social well-being. Theoretically, cultural engagement combines multiple “active ingredients” (i.e., components that make up an activity), including aesthetic engagement, sensory activation, cognitive stimulation, and social interaction. These active ingredients can activate various psychological, biological, social, and behavioral pathways that link cultural engagement to well-being (Fancourt et al., 2021; Warran et al., 2022). These pathways have indeed been empirically tested in intervention and experimental studies. For example, going to concerts can biologically reduce stress hormones (Fancourt & Williamon, 2016) and foster positive emotions (Williams et al., 2023), while visiting museums can have psychological and social benefits through reducing stress and promoting feelings of belonging (Šveb Dragija & Jelinčić, 2022). Evidence from intervention studies has been supported and extended by observational epidemiological studies, which suggest that *day-to-day* cultural engagement can enhance older adults’ well-being. For instance, frequent cultural engagement has been associated with a reduced risk of developing depression (Fancourt & Steptoe, 2019a), lower loneliness (Tymoszuk, Perkins, Fancourt, et al., 2020), and higher well-being (Tymoszuk, Perkins, Spiro, et al., 2020).

However, the existing evidence has limitations, which this study aims to address. First, while the mental and social well-being benefits of cultural engagement have been well-documented, many of these studies have only focused on one mental or social well-being domain. This has made it challenging to compare across studies using different approaches, identify whether cultural engagement impacts extend across different well-being domains, and explore whether different types of cultural activity influence outcomes differently. This study, therefore, examined how cultural engagement was associated with a broad range of mental and social well-being outcomes (i.e., depressive symptoms, anxiety, life satisfaction, quality of life [QoL], having a worthwhile life, happiness, and loneliness) and utilized data collected across 14 years. We examined different cultural engagement activities separately to understand how specific activities are associated with mental and social well-being outcomes. This allows us to identify the “active ingredients” of cultural engagement activities that may be responsible for such associations.

Second, there is a social gradient in cultural engagement among older adults. It varies across sociodemographic groups, and older adults with poorer health are also less likely to engage (Bone et al., 2021; Fluharty et al., 2021). However, most studies have not explored whether such disparities moderate the associations between cultural engagement and mental and social well-being. Understanding whether the benefits of cultural engagement differ across specific population subgroups could support the development of policies and public health interventions that better meet the needs of older adults. Theoretically, it can also increase our understanding of potential moderators of the link between cultural engagement and mental well-being.

Lastly, most existing studies have relied on adjustment for measured confounders. Fixed-effects approaches provide opportunities here by automatically accounting for all time-invariant confounding factors, even if unobserved. Indeed, some confounders of the relationship between cultural engagement and well-being include complex factors relating to demographics, socioeconomic position, personality, past life experiences, and genetics. These may not be adequately assessed within cohort studies, let alone effectively incorporated into analytical models. Additionally, applying generalized method of moments estimators helps to disentange the direction of association through instrumental extensions. However, their use within research on this topic has been limited. This present study addresses these gaps using one of the longest-running longitudinal cohort studies of aging, representative of older adults living in England.

## Method

### Dataset

We analyzed data from the English Longitudinal Study of Ageing (ELSA), which started in 2002/2003 and initially included 12,099 individuals. Respondents aged 50 and over living in private households in England are interviewed every two years in a new wave, with new sample members added to ensure the sample retains its age profile (Steptoe et al., 2013). We used data from wave 2 (2004/2005) through to wave 9 (2018/2019), spanning 14 years, which included questions on cultural engagement and mental and social well-being. Like many longitudinal studies, new questions are regularly added to each new wave. In ELSA, some of the outcomes of interest were only available in later waves. To make full use of the data, we created two analytical samples based on the available data. Sample 1 focused on the outcome measures of depressive symptoms, loneliness, life satisfaction, and QoL, which were assessed in all waves from waves 2 to 9 (*n* = 10,428 participants; *N* = 47,207 observations; average number of waves completed = 4.5). Sample 2 focused on the outcome measures of worthwhile life, happiness, and anxiety, which were only assessed from waves 6 to 9 (*n* = 6,932 participants; *N* = 21,630 observations; average number of waves completed = 3.1).

### Exposure

#### Cultural engagement

Three types of cultural engagement were measured in all eight waves: going to (a) a museum or an art gallery, (b) the theater, a concert, or the opera, and (c) the cinema. For each type of engagement, responses ranged from never engaging to engaging twice a month or more (scale 0–5). As well as being considered independently in sensitivity analyses, these measures of engagement were summed to create an overall cultural engagement score, ranging from 0 to 15, with a higher value indicating more frequent and varied cultural engagement.

### Outcomes

#### Depressive symptoms

Depressive symptoms were measured in all waves using the 8-item version of the Centre for Epidemiological Studies Depression Scale (CES-D), which has been widely used in population surveys (Turvey et al., 1999). The presence of eight symptoms (0 = No, 1 = Yes) was measured (e.g., “You felt depressed?,” “Your sleep was restless?”) and summed to create a total score ranging from 0 to 8, with a higher value indicating more depressive symptoms.

#### Anxiety

Anxiety was measured in waves 6-9 using 1-item from the Office for National Statistics (ONS) well-being measure (ONS, 2018) asking individuals how anxious they felt yesterday. The response scale was rated from 0 to 10, with higher scores indicating higher anxiety.

#### Life satisfaction

Life satisfaction was measured in all waves using the 5-item Satisfaction with Life Scale (SWLS) with items (e.g., “In most ways my life is close to ideal”) scored from 1 to 7 (Diener et al., 1985). Scores were summed, ranging from 5 to 35, with higher scores indicating higher life satisfaction.

#### Quality of life

QoL was measured in all waves using the ELSA-adapted version of the CASP-19 measure, which assesses control, autonomy, self-realization, and pleasure with 19 items (e.g., “How often do you feel full of energy these days”) scored from 0 to 3 (Hyde et al., 2003). All the items are summed, ranging from 0 to 57, with higher scores indicating higher QoL.

#### Having a worthwhile life

Having a worthwhile life was measured in waves 6–9 using 1-item from the ONS well-being measure (ONS, 2018), asking individuals to what extent they feel that the things that they do in their lives are worthwhile. The response scale was rated from 0 to 10, with higher scores indicating higher feelings of having a worthwhile life.

#### Happiness

Happiness was measured in waves 6–9 using 1-item from the ONS well-being measure (ONS, 2018) asking individuals how happy they felt yesterday. The response scale was rated from 0 to 10, with higher scores indicating higher happiness.

#### Loneliness

Loneliness was measured in all waves using the UCLA 3-item Loneliness Scale, validated for use in population surveys (Hughes et al., 2004). The three items (e.g., “How often do you feel lonely?”), scored from 1 to 3, were summed to create a score ranging from 3 to 9, with higher scores indicating higher levels of loneliness.

### Time-Varying Covariates

A set of six time-varying variables that might confound the observed associations between cultural engagement and mental and social well-being was identified from the existing literature (Dahlberg et al., 2021; Fluharty et al., 2021). These included age (continuous in years), wealth (total net nonpension wealth in quintiles), home ownership (not a homeowner, homeowner), employment status (not employed, employed, retired), living with a partner (no, yes), and long-standing illness, disability or infirmity, hereafter titled “health condition” (no illness, nonlimiting illness, limiting illness).

### Statistical Analyses

#### Main analyses

Fixed-effects regression was applied in seven separate models to explore the longitudinal associations between cultural engagement and each of the seven outcomes. Fixed-effects regression assesses whether changes in an exposure (cultural engagement) are associated with changes in an outcome (mental and social well-being). The regression estimates within-individual variation, meaning individuals are compared with themselves over time. Therefore, models automatically control for all individual observed and unobserved time-invariant factors (e.g., stable characteristics such as ethnicity, past life experiences, past mental health and medical history, social class, and genetics; Allison, 2009). Additionally, important observed time-varying confounders were controlled for, and models were built sequentially based on our directed acyclic graphs (DAGs) (Tennant et al., 2021), which mapped the direction of effects between covariates, the exposure, and outcomes (Supplementary Figure 1). Model 0 was unadjusted. In model 1, we adjusted for age, wealth, and home ownership. In model 2, we additionally adjusted for employment status and living with a partner. In model 3, we additionally adjusted for the presence of a health condition. Having a health condition was added in a separate final stage because it may lie on the causal pathway between cultural engagement and mental and social well-being.

#### Sensitivity analyses

To check the robustness of the results, we ran three sensitivity analyses. The first explored how different cultural engagement activities (i.e., museum/gallery, theater/concert/opera, and cinema) were associated with the outcomes, so we repeated the main analyses with each of the three individual items as the exposures.

Second, we ran exploratory analyses to see whether any longitudinal associations from the main analyses varied depending on respondents’ sociodemographic and health characteristics. We therefore included interactions with overall cultural engagement for (a) gender (male, female), (b) baseline age group (50–64 years, 65–79, 80 and above), (c) living with a partner (no, yes), (d) retirement status (not retired, retired), (e) degree status (no degree, degree), and (f) health status (no illness, illness [limiting or nonlimiting]). Interactions are presented as estimates in tables and in margins plots to show predicted values of the outcomes according to cultural engagement levels for each subgroup. All interactions were run in separate fully adjusted models. However, as some interaction variables were derived from the time-varying covariates, these original variables were removed from the model as covariates in specific interaction analyses. For example, the original continuous age variable was removed when running interactions with age groups; the original employment variable was removed when running interactions with retirement status; and the original health condition variable (as three categories) was removed when running interactions with the two-category health status variable. Due to data availability, we could only create a gender variable based on a binary construct of sex. More needs to be done to represent gender identities in cohort studies (Hanes & Clouston, 2021).

Third, given that in fixed-effects regression it is hard to assess the temporal direction of the association between cultural engagement and mental and social well-being measured at the same time, to explore whether cultural engagement might be causally associated with mental and social well-being outcomes we used a generalized method of moments (GMM) estimator, which is an approach that has been used in the literature to explore directionality and improve causal inference (Bone et al., 2022). Using the Stata command “xtabond2,” we applied a two-step system GMM estimator with Windmeijer correction (Roodman, 2009). This estimator uses lagged levels and lagged differences of the outcome variable as instruments in the model (Arellano & Bond, 1991; Blundell & Bond, 1998). The inclusion of time-lagged outcomes into the model takes account of past changes in the outcomes, allowing estimation of how changes in cultural engagement affect changes in outcomes, thus helping to reduce reverse causality. We also treated the exposure (cultural engagement) and time-varying confounders as endogenous variables in the model and included an exogenous dummy variable for wave to improve the model estimation. This approach could only be applied in Sample 1 as Sample 2 (which had outcomes of worthwhile life, happiness, and anxiety) did not have sufficient waves of data. We tested 1, 2, 3, and 4 lags for each outcome (due to the number of waves, we could only test for a maximum of 4 lags). We tested assumptions for autocorrelation (first-order and second-order serial correlations between error terms), difference-in-Hansen tests of exogeneity of instruments (lags need to be uncorrelated with error terms), and Hansen test of over-identification. The number of lags in each outcome model varied dependent on all assumptions being met, and these models were used to establish whether cultural engagement was associated with the outcomes (Supplementary Table 8). All analyses were performed in Stata v.18.

## Results

### Baseline Descriptives

In Sample 1, the mean age was 61.9 years (standard deviation [*SD*] = 9.0), 55% were female, and the mean cultural engagement score was 4.2 (*SD* = 3.2; Table 1). The mean score for depressive symptoms was 1.4 (*SD* = 1.9), 4.1 (*SD* = 1.5) for loneliness, 25.5 (*SD* = 6.4) for life satisfaction, and 42.0 (*SD* = 8.8) for QoL.

**Table 1.** Baseline Descriptives of Analytical Samples

Variable	Sample 1 (*n* = 10,428)		Sample 2 (*n* = 6,932)	
	Mean (*SD*), range	%	Mean (*SD*), range	%
**Age**	61.9 (9.0), 50–99		65.2 (8.7), 50–99	
**Cultural engagement**	4.2 (3.2), 0–15		4.5 (3.2), 0–15	
**Depressive symptoms**	1.4 (1.9), 0–8			
**Loneliness**	4.1 (1.5), 3–9			
**Life satisfaction**	25.5 (6.4), 5–35			
**Quality of life**	42.0 (8.8), 0–57			
**Worthwhile life**			7.5 (2.1), 0–10	
**Happiness**			7.3 (2.2), 0–10	
**Anxiety**			2.0 (2.5), 0–10	
**Gender**				
Male		45.4		44.6
Female		54.6		55.4
**Ethnicity**				
Does not identify as White		2.6		2.7
Identifies as White		97.4		97.3
**Wealth**				
1—lowest quintile		18.8		16.0
2		23.7		18.3
3		21.3		19.9
4		20.0		22.4
5—highest quintile		16.3		23.4
**Education**				
No qualification		25.7		18.0
NVQ1/CSE		4.2		3.6
NVQ2/GCE O-level		20.4		20.7
NVQ3/GCE A-level		8.7		9.6
Higher ed. below degree		14.3		14.7
NVQ4/NVQ5/degree		18.6		20.7
Foreign/other		8.2		12.7
**Home ownership**				
Yes		55.7		66.8
No		44.3		33.2
**Employment**				
Not employed		12.5		8.7
Employed		49.6		39.4
Retired		37.9		51.9
**Living with partner**				
No		24.3		24.2
Yes		75.7		75.8
**Health condition**				
No		48.2		47.9
Yes, not limiting		22.1		22.0
Yes, limiting		29.7		30.1

*Notes*: CSE = Certificate of Secondary Education; GCE = General Certificate of Education; NVQ = National Vocational Qualification; *SD* = standard deviation. Baseline wave varied depending on when participants entered the study. Gender, ethnicity, and education were treated as time-invariant variables so were not included in analyses but they are reported here descriptively for the analytical samples.

In Sample 2, the mean age was 65.2 years (*SD* = 8.7), 55% were female, and the mean cultural engagement score was 4.5 (*SD* = 3.2). The mean score for worthwhile life was 7.5 (*SD* = 2.1), 7.3 (*SD* = 2.2) for happiness, and 2.0 (*SD* = 2.5) for anxiety. Descriptives of the overall mean, overall *SD*, between-individual *SD*, and within-individual *SD* are presented in Supplementary Table 1 in Supplementary Material.

#### Main analyses

**Figure 1.**
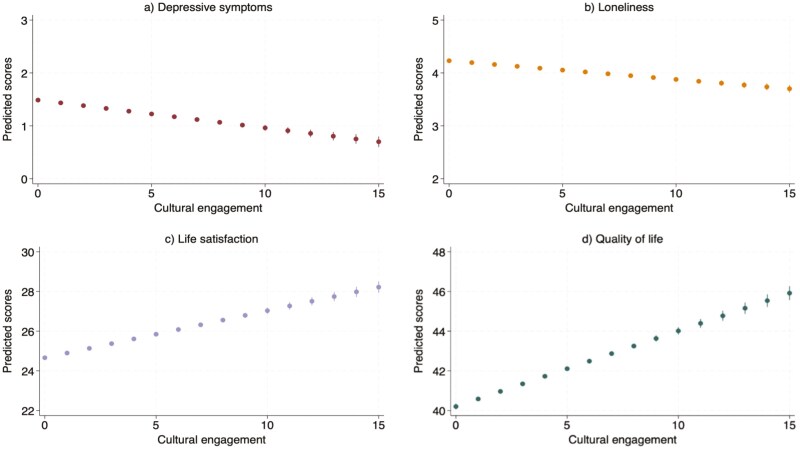
Fixed-effects models estimating the longitudinal associations between cultural engagement and outcomes in fully adjusted models (Sample 1). The sample size is *n* = 10,428. These estimates present within-person variations, showing the longitudinal associations between changes in cultural engagement and changes in the outcomes. Fully adjusted models controlled for age, wealth, home ownership, employment status, living with a partner, and health condition.

**Figure 2.**
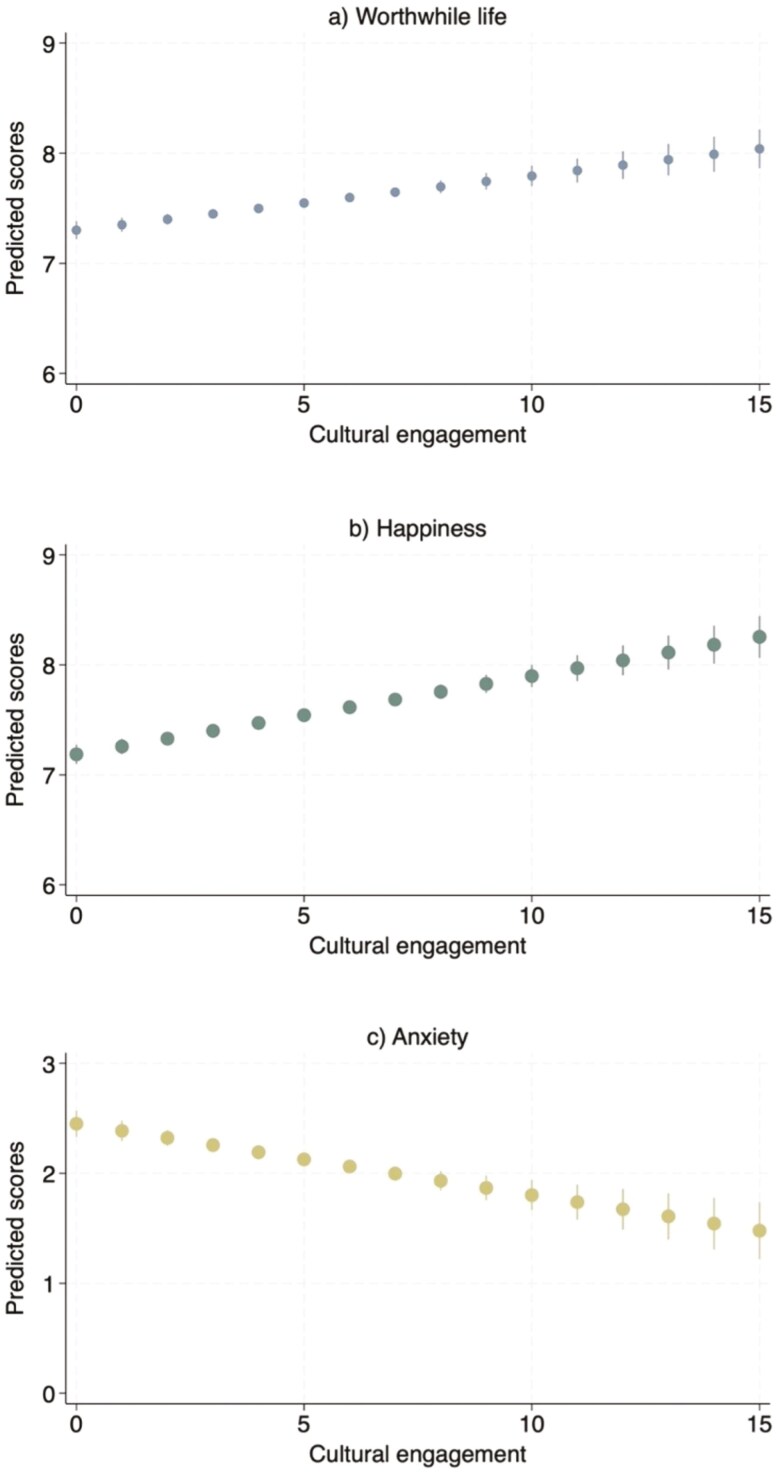
Fixed-effects models estimating the longitudinal associations between cultural engagement and outcomes in fully adjusted models (Sample 2). The sample size is *n* = 6,932. These estimates present within-person variations, showing the longitudinal associations between changes in cultural engagement and changes in the outcomes. Fully adjusted models controlled for age, wealth, home ownership, employment status, living with a partner, and health condition.

After adjusting for all covariates, increases in overall cultural engagement were associated with decreases in depressive symptoms (*B* = −0.05, CI-95% = −0.06, −0.04, *p* < .001), anxiety (*B* = −0.06, CI-95% = −0.09, −0.04, *p* < .001), and loneliness (*B* = −0.04, CI-95% = −0.04, −0.03, *p* < .001), and increases in life satisfaction (*B* = 0.24, CI-95% = 0.21, 0.26, *p* < .001), QoL (*B* = 0.38, CI-95% = 0.35, 0.41, *p* < .001), having a worthwhile life (*B* = 0.05, CI-95% = 0.03, 0.07, *p* < .001), and happiness (*B* = 0.07, CI-95% = 0.05, 0.09, *p* < .001; Figures 1 and 2; Supplementary Table 2).

#### Sensitivity analyses

##### Types of cultural engagement

Every few months or more, 17.4%–19.7% of individuals visited a museum/gallery, 24%–25.8% visited the theater/concert/opera, and 23.4%–25.4% visited the cinema (Supplementary Table 3). When exploring each cultural activity individually, more frequent museum/gallery visits were associated with decreased depressive symptoms, loneliness, and anxiety, and increased life satisfaction, QoL, having a worthwhile life, and happiness (Supplementary Tables 4 and 5). A similar pattern was observed for theater/concert/opera visits and to some extent for cinema visits. Some of these associations showed a dose-response relationship, with more frequent engagement associated with larger changes in outcomes. However, associations were less consistent for anxiety and for cinema visits with having a worthwhile life.

##### Interactions

Exploratory analyses indicated that the longitudinal associations between overall cultural engagement and mental and social well-being outcomes varied across different population subgroups. For depressive symptoms, the association with cultural engagement was stronger for individuals who were female, living without a partner, and who had an illness (Supplementary Table 6, Supplementary Figures 3–5). For loneliness, the association was stronger for those who were female and living without a partner (Supplementary Table 6, Supplementary Figures 3 and 4). For life satisfaction and QoL, the associations were stronger in those who were female, of older age, living without a partner, and who had an illness (Supplementary Table 6, Supplementary Figures 2–5). For having a worthwhile life, the association was stronger in those who were living without a partner (Supplementary Table 7, Supplementary Figure 4). For anxiety, the association was stronger in those aged between 65 and 79 years and with an illness (Supplementary Table 7, Supplementary Figures 2 and 5). There were no interactions with happiness.

##### Directionality of the associations

After accounting for all covariates and previous levels of the outcomes, there was evidence that increases in cultural engagement were associated with decreases in depressive symptoms (*B* = −0.06, CI-95% = −0.11, −0.01, *p* = .010; Supplementary Table 8).

An association between cultural engagement and QoL was also observed when running models with 1–3 lags (*B* = 0.27, CI-95% = 0.09, 0.45, *p* = .003). However, there was no association when including four lags in the model (*B* = 0.13, CI-95% = −0.07, 0.34, *p* = .211). Importantly, the model assumptions for the QoL findings were not met for any of the models; hence, the results require careful interpretation and remain unclear.

Cultural engagement was not associated with loneliness or life satisfaction in the models where assumptions were met.

## Discussion

This paper explored the longitudinal associations between cultural engagement and mental and social well-being outcomes in older adults using data spanning up to 14 years. The main findings indicate that increases in cultural engagement were associated with decreases in depressive symptoms, anxiety, and loneliness, as well as increases in life satisfaction, QoL, feelings of having a worthwhile life, and happiness. Associations continued to be shown when exploring cultural activities separately, especially when visiting a museum/gallery or the theater/concert/opera. Exploring the direction of these associations, increases in cultural engagement were found to be associated with decreases in depressive symptoms. Our study also indicates that some associations might be stronger for certain subgroups of older adults, including females, individuals with a long-standing health condition and living without a partner, and those who are older.

Our main findings mirror the existing literature on the associations between cultural engagement (as defined in this study) with outcomes such as depressive symptoms, loneliness, and life satisfaction in older adults (Fancourt & Steptoe, 2019a; Tymoszuk, Perkins, Fancourt, et al., 2020; Tymoszuk, Perkins, Spiro, et al., 2020). Research on the potential mechanisms linking cultural engagement to mental and social well-being has identified four pathways: psychological, social, biological, and behavioral (Fancourt et al., 2021). Psychologically, cultural engagement could be a strategy to regulate emotions (Fancourt et al., 2019). Socially, cultural engagement is likely to facilitate social contact and interaction, which can aid the development of social identities that can support mental and social well-being (Haslam et al., 2022; Steffens et al., 2021). Biologically, cultural engagement can modulate inflammatory processes (Walker et al., 2019), which are known to be linked to mental well-being (i.e., both depression and mental resilience; Halaris, 2019; Ryan & Ryznar, 2022). Behaviorally, engaging with cultural activities could facilitate being present in the moment, a behavioral technique that can help support mental well-being (Hofmann & Gómez, 2017). However, our results provide an important extension of these previous findings. First, they examine the longitudinal associations between cultural engagement and broader psychological outcomes such as anxiety, which has received significantly less research attention in older adults. Second, they take a much more robust account of confounding factors, demonstrating that a relationship is still present even when accounting for complex unmeasured confounders, including demographics, socioeconomic factors, genetics, and past behaviors and health experiences. Third, the results provide novel insight into the temporality of these relationships. In reality, the relationship between a complex psychosocial behavior like cultural engagement and a multifaceted outcome like well-being is likely bidirectional. Indeed, research exploring the motivations for leisure engagement, and the impacts of leisure engagement, supports the idea of reciprocal influence and positive and negative feedback loops between leisure and mental and social well-being (Fancourt & Warran, 2024; Mak et al., 2024). Our temporal analysis showed that increases in cultural engagement were associated with decreases in depressive symptoms, suggesting that this association was not solely due to lower depressive symptoms leading to increased cultural engagement. Our findings are promising as they imply that promoting cultural engagement could encourage a positive feedback loop between engagement and depression levels.

The findings also show that visiting museums/galleries and going to the theater/concert/opera may similarly benefit mental and social well-being, with dose-response relationships according to engagement frequency. However, going to the cinema was slightly less consistently associated with mental and social well-being. The observed associations may be explained by the active ingredients of these different activities (Warran et al., 2022). For example, visiting museums/galleries and the theater/concert/opera may enable older adults to be exposed to a wider range of key ingredients that could support their well-being, such as emotional resonance, sensory activation, cognitive stimulation, social interactions, and live creative and aesthetic experiences, compared to the cinema. Whilst cinema visits may share some of these ingredients, their recorded nature, potential to be more sedentary, and fewer opportunities for social interaction could explain the slight difference in findings. Additionally, screen time (e.g., TV viewing) has been associated with poorer mental health (Wang et al., 2019) and cognitive decline (Fancourt & Steptoe, 2019b), which may also explain these differences. In support of our findings, previous research on cultural engagement has found stronger associations with visiting museums/galleries and going to the theater/concert/opera as health promoting, than for the cinema (Fancourt & Steptoe, 2018). While these differences may indicate residual confounding, as there could be more socioeconomic disparities in visiting museums/galleries and theaters/concert/opera than going to the cinema, given our statistical approach, any time-invariant factors (alongside the measured time-varying factors identified) were taken into account. Nonetheless, future research is needed into other time-varying confounders that could affect cinema attendance differently compared with other cultural activities.

A final notable finding from our exploratory analyses is that the associations between cultural engagement and mental and social well-being were fairly stable across subpopulations, with a few notable differences. In particular, older adults, those who were female, living without a partner, and with a long-standing health condition, appeared to experience greater well-being improvements with more frequent engagement. These results are crucial because these demographics tend to have poorer mental and social well-being than their counterparts (Dahlberg et al., 2021; Hansen & Blekesaune, 2022; Xie et al., 2023), even though they do not necessarily engage less in cultural activities (Bone et al., 2021; Fluharty et al., 2021; Mak et al., 2020). Indeed, the oldest adults are likely to have more health-related concerns (Maresova et al., 2019). Explanations for these findings may be because cultural engagement is a way to regulate emotions, cope with challenges, give meaning, and support well-being (Fancourt & Ali, 2019; Fancourt et al., 2019; Noguchi & Shang, 2023), as well as supporting transitions synonymous with aging, such as reductions in and shifting of social networks (Wrzus et al., 2013). Whilst socioeconomic indicators, such as higher education levels, play an important role in access to cultural engagement (Bone et al., 2021; Fluharty et al., 2021; Mak et al., 2020), the associations between cultural engagement and mental and social well-being did not vary by these indicators in our findings. This suggests that older adults may experience the same mental and social well-being benefits from cultural engagement irrespective of their socioeconomic status.

This paper has several strengths, including using data collected across a 14-year period, using fixed-effects regressions, and applying additional covariate adjustment that allowed us to control for individual characteristics of people which do not change over time (e.g., fixed genetic effects and past experiences) and those which do (e.g., employment and health status) designing our models iteratively using a causal approach. We also ran a number of sensitivity analyses to test the robustness of findings, including GMM estimators, which allowed us to explore directionality. However, there are several limitations to consider. First, although we used a sophisticated analytical technique, more research is needed using other causal inference models. Second, our study has solely focused on receptive/passive forms of cultural engagement, so future research is required to investigate whether we see similar longitudinal associations with active participation in leisure activities, such as participation in performing arts or volunteering. This will provide further insights into the specific active ingredients and mechanisms that may be responsible for different types of leisure and social activities on the mental and social well-being of older adults. It would also be interesting to explore whether such longitudinal (i.e., multiyear) associations are observed in a therapeutic or intervention setting, such as for Museums on Prescription, which connects older adults with poorer health and well-being to museums or galleries (Thomson et al., 2018). Third, mental and social well-being were measured to give a broad picture of individual well-being for older adults, such as through mental health (i.e., depressive symptoms, anxiety), social well-being (i.e., loneliness), and different aspects of subjective well-being including hedonic/experienced well-being (i.e., happiness), hedonic/evaluative well-being (i.e., life satisfaction), and QoL. Future research is encouraged to look at other aspects of social well-being, such as other functional aspects (i.e., social support), structural and quality aspects of social connections (e.g., social isolation and relationship strain, respectively; Holt-Lunstad, 2018), as well as eudaimonic forms of well-being such as autonomy and self-realization (Stone & Mackie, 2013). In ELSA, there is a lack of information on whether cultural activities are done alone or with others. Exploring this would help us understand how these types of engagement may also promote group-level well-being, including social connectedness. Finally, our results may not be generalizable to populations outside of the U.K., so further work to explore cross-country findings is encouraged.

## Conclusion

Our analyses show that increases in cultural engagement are associated with different aspects of mental and social well-being, including decreases in depressive symptoms, anxiety, and loneliness, as well as increases in life satisfaction, QoL, and having a worthwhile life and happiness. Findings were partly driven by two forms of cultural engagement, specifically going to museums/galleries and theater/concerts/opera. Exploring the directionality of these associations, the association between cultural engagement and depressive symptoms persisted even after accounting for prior levels of depressive symptoms. There were some indications that the associations varied across some subgroups of older adults, including those who traditionally have poorer health, experiencing greater benefits from cultural engagement. Overall, our findings have several implications. First, they highlight the importance of ensuring equitable access to cultural engagement for all older adults through funding cultural activities and spaces. Second, our results are of importance to specific schemes supporting older adults, such as through Social Prescribing and Museums on Prescription services that help connect older adults to cultural and community activities.

## Supplementary Material

gbaf074_suppl_Supplementary_Materials_1

## Data Availability

The analysis code is available via OSF (https://osf.io/v6ds3/). The ELSA dataset is publicly available via the UK Data Service (https://beta.ukdataservice.ac.uk/datacatalogue/series/series?id=200011#!/access-data). The research was not preregistered.
