# Supplementary material for: Longitudinal Associations Between Cultural Engagement and Mental and Social Well-Being: A Fixed-Effects Analysis of the English Longitudinal Study of Ageing
Source: J Gerontol B Psychol Sci Soc Sci. 2025 Apr 22;80(7):gbaf074. doi: 10.1093/geronb/gbaf074 (PMC12362352; doi:10.1093/geronb/gbaf074)
Supplement: gbaf074_suppl_Supplementary_Materials_1 [file gbaf074_suppl_supplementary_materials_1.docx]

# ***The Journals of Gerontology, Series B: Psychological Sciences and Social Sciences* Supplementary Material: Finn et al. Longitudinal associations between cultural engagement and mental and social well-being: A fixed-effects analysis of the English Longitudinal Study of Ageing.**

## **Supplementary Table 1.** Descriptives of the overall mean, overall standard deviation (SD), between-individual SD, and within-individual SD of the analytical sample not restricted to baseline.

| **Sample 1** | **Overall mean** | **Overall SD** | **Between-individual SD** | **Within-individual SD** |
| --- | --- | --- | --- | --- |
| Cultural engagement | 4.3 | 3.2 | 3.0 | 1.2 |
| Depressive symptoms | 1.3 | 1.8 | 1.5 | 1.1 |
| Loneliness | 4.1 | 1.5 | 1.3 | 0.8 |
| Life satisfaction | 25.7 | 6.3 | 5.6 | 3.2 |
| Quality of life | 41.9 | 8.7 | 8.0 | 4.0 |
| **Sample 2** | **Overall mean** | **Overall SD** | **Between-individual SD** | **Within-individual SD** |
| Cultural engagement | 4.6 | 3.2 | 3.1 | 1.1 |
| Worthwhile life | 7.5 | 2.1 | 1.8 | 1.1 |
| Happiness | 7.5 | 2.0 | 1.7 | 1.2 |
| Anxiety | 2.2 | 2.5 | 2.0 | 1.7 |

Sample 1 is n=10,428 and Sample 2 is n=6,932.

## **Supplementary Table 2.** Fixed-effects estimates for main analyses across all models (Samples 1 and 2).

|  | **Model 0** | | | **Model 1** | | | **Model 2** | | | **Model 3** | | |
| --- | --- | --- | --- | --- | --- | --- | --- | --- | --- | --- | --- | --- |
|  | **B** | **CI-95%** | **p-value** | **B** | **CI-95%** | **p-value** | **B** | **CI-95%** | **p-value** | **B** | **CI-95%** | **p-value** |
| **Sample 1** |  |  |  |  |  |  |  |  |  |  |  |  |
| Depressive symptoms | -0.05 | -0.06, -0.04 | <.001 | -0.05 | -0.06, -0.04 | <.001 | -0.06 | -0.06, -0.05 | <.001 | -0.05 | -0.06, -0.04 | <.001 |
| Loneliness | -0.03 | -0.04, -0.02 | <.001 | -0.03 | -0.04, -0.02 | <.001 | -0.04 | -0.04, -0.03 | <.001 | -0.04 | -0.04, -0.03 | <.001 |
| Life satisfaction | 0.23 | 0.21, 0.26 | <.001 | 0.23 | 0.21, 0.26 | <.001 | 0.24 | 0.22, 0.27 | <.001 | 0.24 | 0.21, 0.26 | <.001 |
| Quality of life | 0.46 | 0.42, 0.49 | <.001 | 0.40 | 0.37, 0.44 | <.001 | 0.40 | 0.36, 0.43 | <.001 | 0.38 | 0.35, 0.41 | <.001 |
| **Sample 2** |  |  |  |  |  |  |  |  |  |  |  |  |
| Worthwhile life | 0.05 | 0.03, 0.07 | <.001 | 0.05 | 0.03, 0.07 | <.001 | 0.05 | 0.03, 0.07 | <.001 | 0.05 | 0.03, 0.07 | <.001 |
| Happiness | 0.07 | 0.05, 0.09 | <.001 | 0.07 | 0.05, 0.09 | <.001 | 0.07 | 0.05, 0.09 | <.001 | 0.07 | 0.05, 0.09 | <.001 |
| Anxiety | -0.07 | -0.10, -0.05 | <.001 | -0.07 | -0.09, -0.04 | <.001 | -0.07 | -0.09, -0.04 | <.001 | -0.06 | -0.09, -0.04 | <.001 |

Sample 1 is n=10,428 and Sample 2 is n=6,932. Model 0: no time-varying covariates were controlled for. Model 1: adjusted for age, wealth, and home ownership. Model 2: adjusted for employment status and living with a partner. Model 3: adjusted for health condition. B = unstandardised beta estimates relating to a 1-point increase in cultural engagement. CI-95% = 95% confidence intervals.

## **Supplementary Table 3.** Descriptives of the frequency of individual cultural activities (Samples 1 and 2).

| **Sample 1** | | **Sample 2** | |
| --- | --- | --- | --- |
| **Percentage (%)** | | | |
| **Museum/Gallery** | | | |
| Never | 34.2 | Never | 30.9 |
| Less than once a year | 27.5 | Less than once a year | 27.4 |
| Once or twice a year | 20.9 | Once or twice a year | 22.0 |
| Every few months | 12.8 | Every few months | 14.5 |
| Once a month or more | 4.6 | Once a month or more | 5.2 |
| **Theatre/Concert/Opera** | | | |
| Never | 29.5 | Never | 25.9 |
| Less than once a year | 21.9 | Less than once a year | 22.6 |
| Once or twice a year | 24.5 | Once or twice a year | 25.8 |
| Every few months | 17.7 | Every few months | 19.4 |
| Once a month or more | 6.3 | Once a month or more | 6.4 |
| **Cinema** | | | |
| Never | 32.8 | Never | 28.6 |
| Less than once a year | 23.8 | Less than once a year | 24.6 |
| Once or twice a year | 20.0 | Once or twice a year | 21.4 |
| Every few months | 16.7 | Every few months | 18.6 |
| Once a month or more | 6.7 | Once a month or more | 6.8 |

Sample 1 is n=10,428 and Sample 2 is n=6,932. Categories of about once a month and twice a month or more were collapsed into one category of monthly or more.

## **Supplementary Table 4.** Fixed-effects analysis in fully-adjusted model: Individual cultural activities (Sample 1).

|  | **Sample 1** | | | | | | | | | |
| --- | --- | --- | --- | --- | --- | --- | --- | --- | --- | --- |
|  | **Museum/Gallery** | | | **Theatre/Concert/Opera** | | | **Cinema** | | | |
|  |  | | | **Depressive symptoms** | | |  | | | |
|  | **B** | **CI-95%** | **p-value** | **B** | **CI-95%** | **p-value** | **B** | **CI-95%** | **p-value** |  |
| Less than once a year | **-0.14** | **-0.19, -0.09** | **<.001** | **-0.15** | **-0.20, -0.10** | **<.001** | **-0.10** | **-0.14, -0.05** | **<.001** |  |
| Once or twice a year | **-0.21** | **-0.27, -0.15** | **<.001** | **-0.20** | **-0.26, -0.15** | **<.001** | **-0.13** | **-0.19, -0.07** | **<.001** |  |
| Every few months | **-0.32** | **-0.39, -0.25** | **<.001** | **-0.27** | **-0.33, -0.20** | **<.001** | **-0.17** | **-0.24, -0.11** | **<.001** |  |
| Once a month or more | **-0.38** | **-0.48, -0.29** | **<.001** | **-0.31** | **-0.40, -0.22** | **<.001** | **-0.24** | **-0.33, -0.16** | **<.001** |  |
|  |  |  |  |  | **Loneliness** |  |  |  |  |  |
|  | **B** | **CI-95%** | **p-value** | **B** | **CI-95%** | **p-value** | **B** | **CI-95%** | **p-value** |  |
| Less than once a year | **-0.09** | **-0.12, -0.06** | **<.001** | **-0.09** | **-0.13, -0.05** | **<.001** | **-0.06** | **-0.09, -0.02** | **0.002** |  |
| Once or twice a year | **-0.12** | **-0.16, -0.08** | **<.001** | **-0.15** | **-0.19, -0.11** | **<.001** | **-0.11** | **-0.15, -0.07** | **<.001** |  |
| Every few months | **-0.15** | **-0.20, -0.10** | **<.001** | **-0.18** | **-0.22, -0.13** | **<.001** | **-0.16** | **-0.21, -0.11** | **<.001** |  |
| Once a month or more | **-0.18** | **-0.25, -0.11** | **<.001** | **-0.21** | **-0.27, -0.14** | **<.001** | **-0.21** | **-0.27, -0.14** | **<.001** |  |
|  | **Life satisfaction** | | | | | | | | | |
|  | **B** | **CI-95%** | **p-value** | **B** | **CI-95%** | **p-value** | **B** | **CI-95%** | **p-value** |  |
| Less than once a year | **0.54** | **0.40, 0.67** | **<.001** | **0.51** | **0.36, 0.65** | **<.001** | **0.47** | **0.33, 0.61** | **<.001** |  |
| Once or twice a year | **0.81** | **0.64, 0.97** | **<.001** | **0.87** | **0.71, 1.03** | **<.001** | **0.76** | **0.59, 0.93** | **<.001** |  |
| Every few months | **1.16** | **0.96, 1.36** | **<.001** | **1.18** | **0.99, 1.37** | **<.001** | **0.94** | **0.74, 1.13** | **<.001** |  |
| Once a month or more | **1.48** | **1.20, 1.76** | **<.001** | **1.41** | **1.15, 1.67** | **<.001** | **1.21** | **0.96, 1.45** | **<.001** |  |
|  | **Quality of life** | | | | | | | | | |
|  | **B** | **CI-95%** | **p-value** | **B** | **CI-95%** | **p-value** | **B** | **CI-95%** | **p-value** |  |
| Less than once a year | **0.89** | **0.72, 1.05** | **<.001** | **0.93** | **0.75, 1.11** | **<.001** | **0.63** | **0.46, 0.80** | **<.001** |  |
| Once or twice a year | **1.40** | **1.20, 1.60** | **<.001** | **1.55** | **1.36, 1.75** | **<.001** | **0.99** | **0.78, 1.20** | **<.001** |  |
| Every few months | **1.80** | **1.56, 2.05** | **<.001** | **1.98** | **1.75, 2.21** | **<.001** | **1.53** | **1.29, 1.76** | **<.001** |  |
| Once a month or more | **2.30** | **1.96, 2.65** | **<.001** | **2.49** | **2.17, 2.80** | **<.001** | **1.79** | **1.49, 2.09** | **<.001** |  |

Sample 1 is n=10,428. The associations were run in fully-adjusted models, controlling for age, wealth, home ownership, employment status, living with a partner, and health condition. B = unstandardised beta estimates relating to a 1-point increase in cultural engagement. CI-95% = 95% confidence intervals. The cultural engagement reference category is Never.

## **Supplementary Table 5.** Fixed-effects analysis in fully-adjusted model: Individual cultural activities (Sample 2).

|  | **Sample 2** | | | | | | | | |
| --- | --- | --- | --- | --- | --- | --- | --- | --- | --- |
|  | **Museum/Gallery** | | | **Theatre/Concert/Opera** | | | **Cinema** | | |
|  |  | | | **Worthwhile life** | | |  | | |
|  | **B** | **CI-95%** | **p-value** | **B** | **CI-95%** | **p-value** | **B** | **CI-95%** | **p-value** |
| Less than once a year | **0.20** | **0.12, 0.29** | **<.001** | **0.11** | **0.02, 0.20** | **0.020** | 0.05 | -0.03, 0.14 | 0.231 |
| Once or twice a year | **0.25** | **0.15, 0.35** | **<.001** | **0.26** | **0.16, 0.36** | **<.001** | **0.13** | **0.03, 0.24** | **0.014** |
| Every few months | **0.32** | **0.19, 0.44** | **<.001** | **0.25** | **0.13, 0.36** | **<.001** | 0.11 | -0.01, 0.23 | 0.071 |
| Once a month or more | **0.41** | **0.24, 0.58** | **<.001** | **0.25** | **0.09, 0.42** | **0.002** | **0.22** | **0.06, 0.37** | **0.006** |
|  |  |  |  |  | **Happiness** |  |  |  |  |
|  | **B** | **CI-95%** | **p-value** | **B** | **CI-95%** | **p-value** | **B** | **CI-95%** | **p-value** |
| Less than once a year | **0.13** | **0.04, 0.22** | **0.005** | 0.09 | -0.00, 0.19 | 0.063 | 0.08 | -0.02, 0.17 | 0.125 |
| Once or twice a year | **0.22** | **0.11, 0.33** | **<.001** | **0.22** | **0.11, 0.33** | **<.001** | **0.18** | **0.07, 0.30** | **0.002** |
| Every few months | **0.40** | **0.27, 0.54** | **<.001** | **0.32** | **0.19, 0.45** | **<.001** | **0.22** | **0.09, 0.35** | **0.001** |
| Once a month or more | **0.64** | **0.46, 0.82** | **<.001** | **0.36** | **0.19, 0.54** | **<.001** | **0.28** | **0.11, 0.44** | **0.001** |
|  | **Anxiety** | | | | | | | | |
|  | **B** | **CI-95%** | **p-value** | **B** | **CI-95%** | **p-value** | **B** | **CI-95%** | **p-value** |
| Less than once a year | -0.08 | -0.20, 0.05 | 0.230 | -0.10 | -0.24, 0.03 | 0.126 | -0.02 | -0.15, 0.12 | 0.818 |
| Once or twice a year | -0.15 | -0.30, 0.00 | 0.054 | **-0.27** | **-0.42, -0.12** | **<.001** | -0.14 | -0.30, 0.02 | 0.089 |
| Every few months | **-0.23** | **-0.41, -0.04** | **0.015** | **-0.35** | **-0.53, -0.18** | **<.001** | **-0.22** | **-0.39, -0.04** | **0.018** |
| Once a month or more | **-0.31** | **-0.56, -0.06** | **0.015** | **-0.52** | **-0.75, -0.28** | **<.001** | **-0.23** | **-0.45, -0.00** | **0.048** |

Sample 2 is n=6,932. The associations were run in fully-adjusted models, controlling for age, wealth, home ownership, employment status, living with a partner, and health condition. B = unstandardised beta estimates relating to a 1-point increase in cultural engagement. CI-95% = 95% confidence intervals. The cultural engagement reference category is Never.

## **Supplementary Table 6.** Fixed-effects analysis in fully-adjusted model: Interaction estimates for depressive symptoms, loneliness, life satisfaction and quality of life outcomes (Sample 1).

|  | **Depressive symptoms** | | | **Loneliness** | | | | **Life satisfaction** | | | **Quality of life** | | |
| --- | --- | --- | --- | --- | --- | --- | --- | --- | --- | --- | --- | --- | --- |
|  | **B** | **CI-95%** | **p-value** | **B** | **CI-95%** | **p-value** | **B** | | **CI-95%** | **p-value** | **B** | **CI-95%** | **p-value** |
| Cultural engagement | -0.04 | -0.05, -0.03 | <.001 | -0.02 | -0.03, -0.01 | <.001 | 0.20 | | 0.16, 0.24 | <.001 | 0.36 | 0.31, 0.41 | <.001 |
| Cultural engagement*Female | **-0.02** | **-0.04, -0.00** | **0.013** | **-0.02** | **-0.04,-0.01** | **0.001** | **0.06** | | **0.01, 0.12** | **0.019** | **0.10** | **0.04, 0.17** | **0.002** |
| Cultural engagement | -0.05 | -0.06, -0.04 | <.001 | -0.03 | -0.04, -0.02 | <.001 | 0.20 | | 0.17, 0.23 | <.001 | 0.32 | 0.28, 0.36 | <.001 |
| Cultural engagement*65-79 | -0.01 | -0.03, 0.01 | 0.353 | -0.00 | -0.02, 0.01 | 0.792 | **0.09** | | **0.03, 0.15** | **0.002** | **0.27** | **0.20, 0.34** | **<.001** |
| Cultural engagement*80+ | -0.00 | -0.06, 0.06 | 1.000 | -0.02 | -0.06, 0.02 | 0.431 | **0.20** | | **0.04, 0.36** | **0.015** | **0.45** | **0.25, 0.65** | **<.001** |
| Cultural engagement | -0.09 | -0.10, -0.07 | <.001 | -0.06 | -0.07, -0.05 | <.001 | 0.29 | | 0.25, 0.34 | <.001 | 0.49 | 0.44, 0.54 | <.001 |
| Living with partner | -0.96 | -1.06, -0.85 | <.001 | -1.24 | -1.32, -1.17 | <.001 | 2.73 | | 2.42, 3.03 | <.001 | 1.34 | 0.97, 1.71 | <.001 |
| Cultural engagement*Partner | **0.05** | **0.03, 0.06** | **<.001** | **0.03** | **0.02, 0.05** | **<.001** | **-0.08** | | **-0.13, -0.03** | **0.001** | **-0.15** | **-0.21, -0.10** | **<.001** |
| Cultural engagement | -0.05 | -0.06, -0.04 | <.001 | -0.04 | -0.04, -0.03 | <.001 | 0.23 | | 0.20, 0.27 | <.001 | 0.36 | 0.32, 0.40 | <.001 |
| Retired | -0.07 | -0.13, -0.01 | 0.028 | -0.05 | -0.09, -0.00 | 0.043 | 0.48 | | 0.30, 0.66 | <.001 | 0.80 | 0.58, 1.02 | <.001 |
| Cultural engagement*Retired | -0.00 | -0.01, 0.01 | 0.478 | 0.00 | -0.01, 0.01 | 0.705 | 0.00 | | -0.03, 0.03 | 0.862 | 0.03 | -0.00, 0.07 | 0.084 |
| Cultural engagement | -0.05 | -0.06, -0.04 | <.001 | -0.03 | -0.04, -0.03 | <.001 | 0.24 | | 0.21, 0.27 | <.001 | 0.39 | 0.35, 0.43 | <.001 |
| Cultural engagement*Degree | -0.00 | -0.03, 0.02 | 0.807 | -0.01 | -0.03, 0.01 | 0.197 | -0.00 | | -0.07, 0.06 | 0.963 | -0.04 | -0.12, 0.04 | 0.318 |
| Cultural engagement | -0.04 | -0.05, -0.03 | <.001 | -0.03 | -0.04, -0.03 | <.001 | 0.22 | | 0.19, 0.26 | <.001 | 0.34 | 0.30, 0.38 | <.001 |
| Has illness | 0.25 | 0.19, 0.31 | <.001 | 0.06 | 0.02, 0.10 | 0.005 | -0.46 | | -0.63, -0.29 | <.001 | -1.28 | -1.49,-1.07 | <.001 |
| Cultural engagement*Illness | **-0.03** | **-0.04, -0.02** | **<.001** | -0.00 | -0.01, 0.00 | 0.212 | **0.03** | | **0.00, 0.06** | **0.045** | **0.09** | **0.06, 0.13** | **<.001** |

The sample size is n=10,428. The interactions were run in fully-adjusted models, controlling for age, wealth, home ownership, employment status, living with partner, and health condition. Where interaction variables (i.e., age groups, retirement, and health status) were derived from the time-varying covariates, these original covariates were removed from the model in specific interaction analyses. B = unstandardised beta estimates relating to a 1-point increase in cultural engagement. CI-95% = 95% confidence intervals. Reference groups (in order) were male, aged 50-64, living without a partner, not retired, no degree, and no illness. Variables treated as time-invariant were omitted automatically from the models (gender, age, and degree status).

## **Supplementary Table 7.** Fixed-effects analysis in fully-adjusted model: Interaction estimates for worthwhile life, happiness, and anxiety outcomes (Sample 2).

|  | **Worthwhile life** | | | **Happiness** | | | **Anxiety** | | |
| --- | --- | --- | --- | --- | --- | --- | --- | --- | --- |
|  | **B** | **CI-95%** | **p-value** | **B** | **CI-95%** | **p-value** | **B** | **CI-95%** | **p-value** |
| Cultural engagement | 0.06 | 0.03, 0.08 | <.001 | 0.08 | 0.06, 0.11 | <.001 | -0.06 | -0.10, -0.02 | 0.002 |
| Cultural engagement*Female | -0.01 | -0.05, 0.02 | 0.459 | -0.02 | -0.06, 0.01 | 0.227 | -0.01 | -0.06, 0.04 | 0.791 |
| Cultural engagement | 0.04 | 0.02, 0.06 | <.001 | 0.07 | 0.05, 0.09 | <.001 | -0.05 | -0.08, -0.02 | <.001 |
| Cultural engagement*65-79 | 0.02 | -0.02, 0.06 | 0.254 | -0.03 | -0.08, 0.01 | 0.140 | **-0.09** | **-0.15, -0.03** | **0.004** |
| Cultural engagement*80+ | 0.07 | -0.12, 0.27 | 0.470 | 0.07 | -0.14, 0.28 | 0.505 | 0.07 | -0.22, 0.36 | 0.633 |
| Cultural engagement | 0.10 | 0.07, 0.13 | <.001 | 0.08 | 0.05, 0.11 | <.001 | -0.06 | -0.10, -0.02 | 0.002 |
| Living with partner | 0.92 | 0.71, 1.13 | <.001 | 0.59 | 0.36, 0.81 | <.001 | -0.02 | -0.33, 0.29 | 0.910 |
| Cultural engagement*Partner | **-0.07** | **-0.10, -0.04** | **<.001** | -0.02 | -0.05, 0.02 | 0.319 | -0.00 | -0.05, 0.04 | 0.932 |
| Cultural engagement | 0.05 | 0.03, 0.08 | <.001 | 0.07 | 0.04, 0.09 | <.001 | -0.06 | -0.10, -0.03 | <.001 |
| Retired | 0.10 | -0.02, 0.23 | 0.114 | 0.10 | -0.03, 0.24 | 0.140 | -0.33 | -0.51, -0.14 | 0.001 |
| Cultural engagement*Retired | -0.01 | -0.03, 0.01 | 0.412 | 0.01 | -0.02, 0.03 | 0.582 | -0.00 | -0.03, 0.03 | 0.898 |
| Cultural engagement | 0.05 | 0.03, 0.07 | <.001 | 0.08 | 0.06, 0.10 | <.001 | -0.06 | -0.08, -0.03 | <.001 |
| Cultural engagement*Degree | -0.00 | -0.04, 0.04 | 0.983 | -0.03 | -0.07, 0.02 | 0.234 | -0.03 | -0.09, 0.02 | 0.240 |
| Cultural engagement | 0.04 | 0.02, 0.06 | <.001 | 0.07 | 0.05, 0.09 | <.001 | -0.04 | -0.07, -0.01 | 0.003 |
| Has illness | -0.15 | -0.26,-0.04 | 0.007 | -0.04 | -0.16, 0.08 | 0.505 | 0.22 | 0.06, 0.38 | 0.006 |
| Cultural engagement*Illness | 0.01 | -0.01, 0.03 | 0.227 | 0.01 | -0.01, 0.03 | 0.401 | **-0.04** | **-0.07, -0.01** | **0.003** |

The sample size is n=6,932. The interactions were run in fully-adjusted models, controlling for age, wealth, home ownership, employment status, living with a partner, and health condition. Where interaction variables (i.e., age groups, retirement, and health status) were derived from the time-varying covariates, these original covariates were removed from the model in specific interaction analyses. B = unstandardised beta estimates relating to a 1-point increase in cultural engagement. CI-95% = 95% confidence intervals. Reference groups (in order) were male, aged 50-64, living without a partner, not retired, no degree, and no illness. Variables treated as time-invariant were omitted automatically from the models (gender, age, and degree status).

## **Supplementary Table 8.** System Generalised Method of Moments (GMM) estimates and assumptions (Sample 1).

| **Depressive symptoms** | | | |
| --- | --- | --- | --- |
|  | **3 lags** | | |
|  | **B** | **CI-95%** | **p-value** |
| **Cultural engagement** | -0.06 | -0.11, -0.01 | 0.010 |
| Autocorrelation (AR1) |  |  | <.001 |
| Autocorrelation (AR2) |  |  | 0.882 |
| Hansen test for overidentification of instruments |  |  | 0.230 |
| Exogeneity of instruments |  |  | 0.070 |
| **Loneliness** | | | |
|  | **4 lags** | |  |
|  | **B** | **CI-95%** | **p-value** |
| **Cultural engagement** | -0.04 | -0.09, 0.01 | 0.101 |
| Autocorrelation (AR1) |  |  | <.001 |
| Autocorrelation (AR2) |  |  | 0.535 |
| Hansen test for overidentification of instruments |  |  | 0.306 |
| Exogeneity of instruments |  |  | 0.259 |
| **Life satisfaction** | | | |
|  | **3 lags** | | |
|  | **B** | **CI-95%** | **p-value** |
| **Cultural engagement** | 0.10 | -0.04, 0.24 | 0.169 |
| Autocorrelation (AR1) |  |  | <.001 |
| Autocorrelation (AR2) |  |  | 0.124 |
| Hansen test for overidentification of instruments |  |  | 0.232 |
| Exogeneity of instruments |  |  | 0.054 |
| **Quality of life** | | | |
|  | **3 lags** | | |
|  | **B** | **CI-95%** | **p-value** |
| **Cultural engagement** | 0.27 | 0.09, 0.45 | 0.003 |
| Autocorrelation (AR1) |  |  | <.001 |
| Autocorrelation (AR2) |  |  | 0.547 |
| Hansen test for overidentification of instruments |  |  | 0.083 |
| Exogeneity of instruments |  |  | <.001 |

Green depicts where model assumptions were met for first-order serial correlations, second-order serial correlations, overidentification restrictions, and exogeneity of instruments. The separate analyses of cultural engagement on the four outcomes differ regarding the number of lags in their models. The number of lags in each model was chosen based on where the first model assumptions were met when testing 1 to 4 lags (please see ‘Directionality of the association’ in the manuscript for reporting of QoL). Depressive symptoms: Lags 1-3, n=5,055, waves=1-5. Loneliness: Lags 1-4, n=3,512; waves=1-4. Life satisfaction: Lags 1-3, n=5,055; waves 1-5. Quality of life: Lags 1-3, n=5,055; waves 1-5. To ensure a large sample size, the models were not restricted to having complete data for other variables that were not included in the model (i.e., when we estimated cultural engagement on depressive symptoms, we did not restrict to those who also had life satisfaction data).

## **Supplementary Figure 1.** Directed acyclic graphs (DAGs) for model building using time-varying covariates.

**
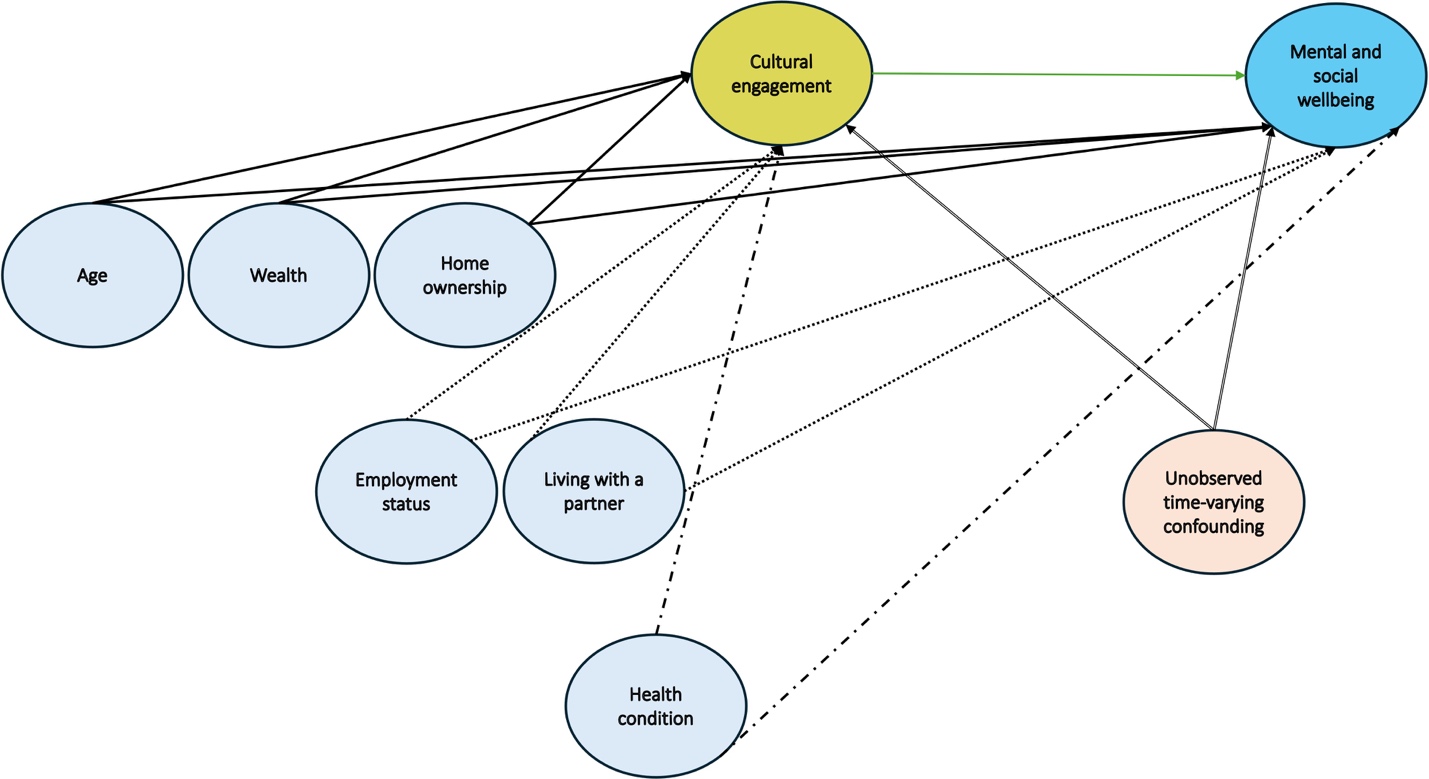
**

This DAG shows the focal relationship between cultural engagement and mental and social well-being. The black arcs represent the certainty of the adjusted variables (age, wealth, home ownership, employment status, living with a partner, and health condition) being confounders between cultural engagement and mental and social well-being. The more spacing/dashed the black lines (rather than solid) the more uncertainty about that variable as a confounder as it could potentially lie on the causal pathway between cultural engagement and mental and social well-being.

Alt text:

A directed acyclic graph (DAG) showing the relationships between the adjusted covariates in relation to the cultural engagement exposure and mental and social wellbeing outcomes, which aided the building of the iterative covariate models.

## **Supplementary Figure 2.** Interactions graphs for age groups.


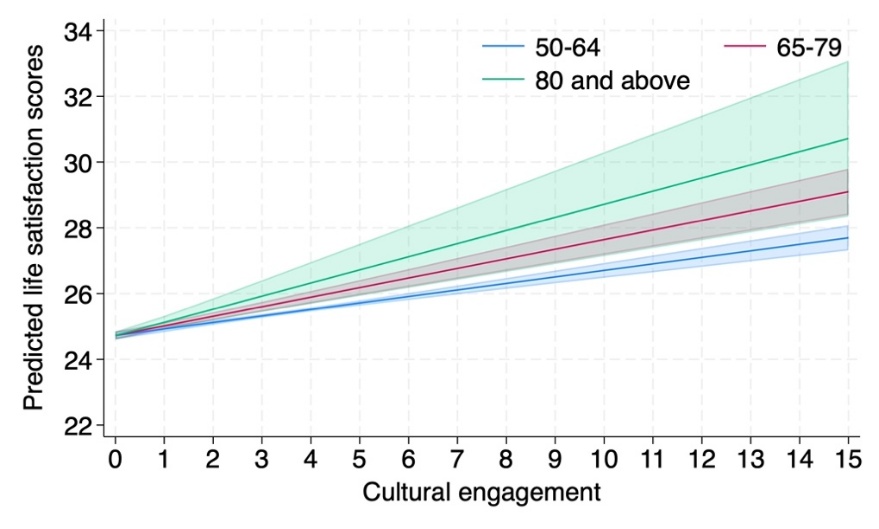


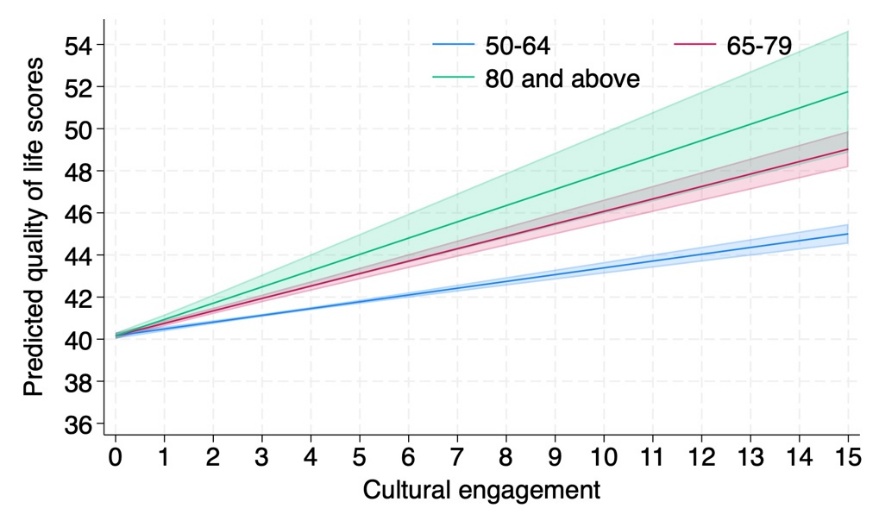


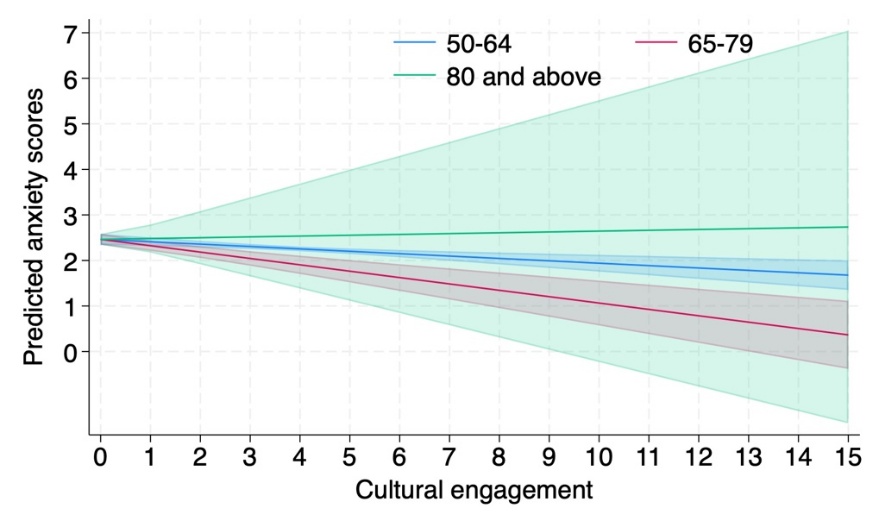


Sample 1 is n=10,428 and Sample 2 is n=6,932. These estimates are from fixed-effects models and present within-person variations, showing the longitudinal associations between changes in cultural engagement and changes in the outcomes. Fully-adjusted models controlled for wealth, home ownership, employment status, living with a partner and health condition.

Alt Text: Scatterplot graphs showing age moderating the associations between cultural engagement with life satisfaction, quality of life and anxiety.

## **Supplementary Figure 3.** Interactions graphs for gender.


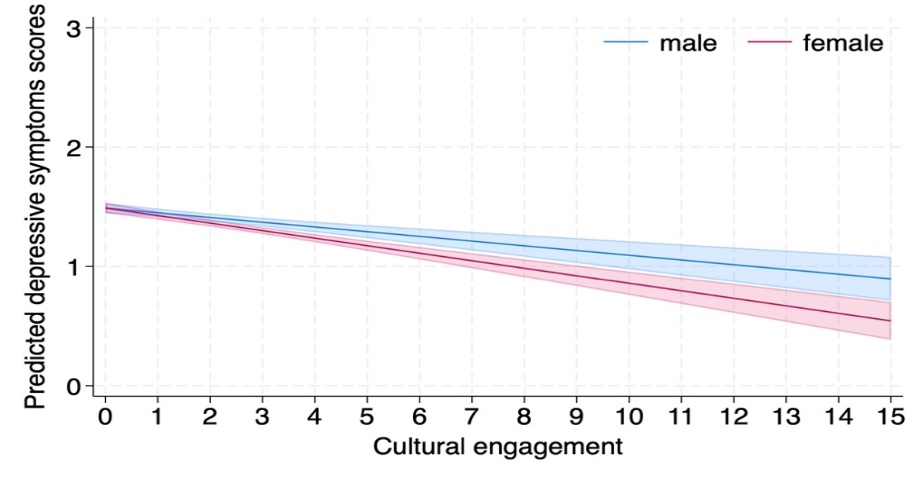

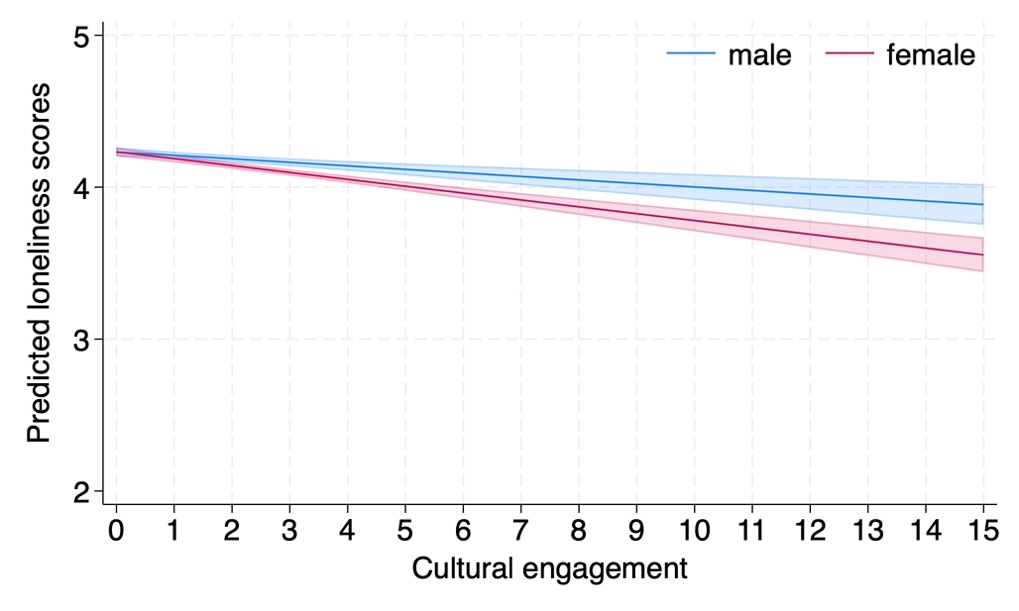

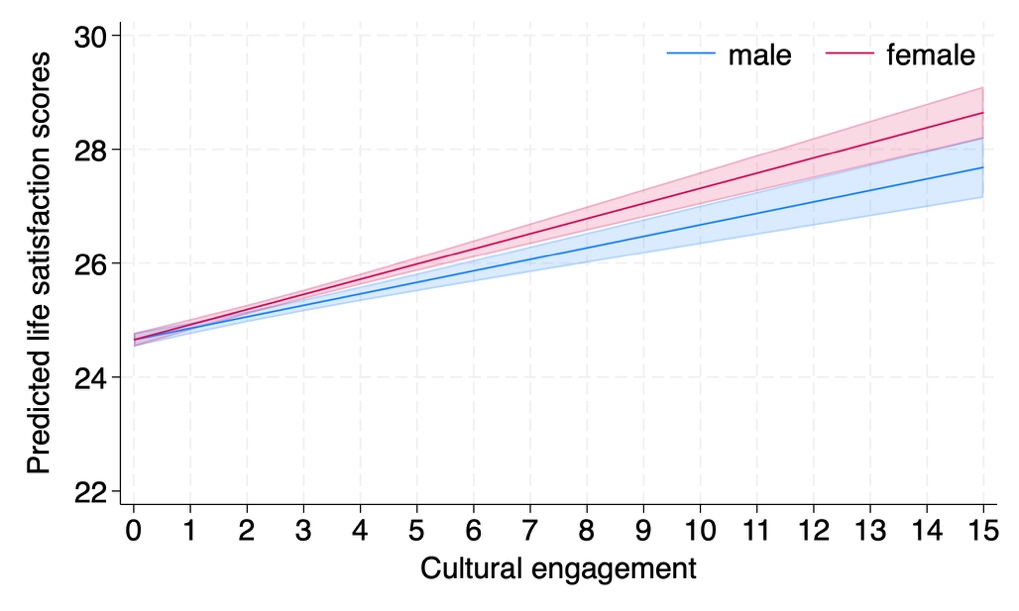

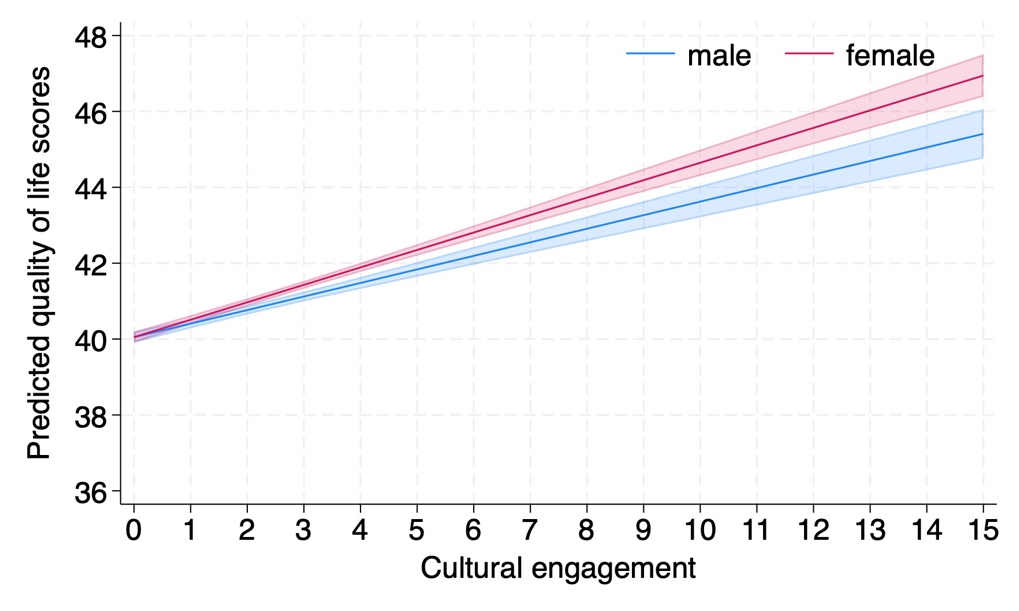


Sample 1 is n=10,428 and Sample 2 is n=6,932. These estimates are from fixed-effects models and present within-person variations, showing the longitudinal associations between changes in cultural engagement and changes in the outcomes. Fully-adjusted models controlled for age, wealth, home ownership, employment status, living with a partner and health condition.

Alt Text: Scatterplot graphs showing gender moderating the associations between cultural engagement with depressive symptoms, loneliness, life satisfaction and quality of life.

## **Supplementary Figure 4.** Interactions graphs for living with a partner.

Sample 1 is n=10,428 and Sample 2 is n=6,932. These estimates are from fixed-effects models and present within-person variations, showing the longitudinal associations between changes in cultural engagement and changes in the outcomes. Fully-adjusted models controlled for age, wealth, home ownership, employment status, and health condition.


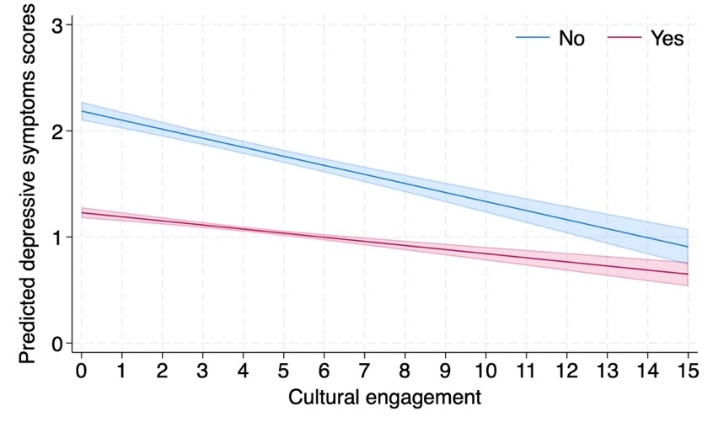

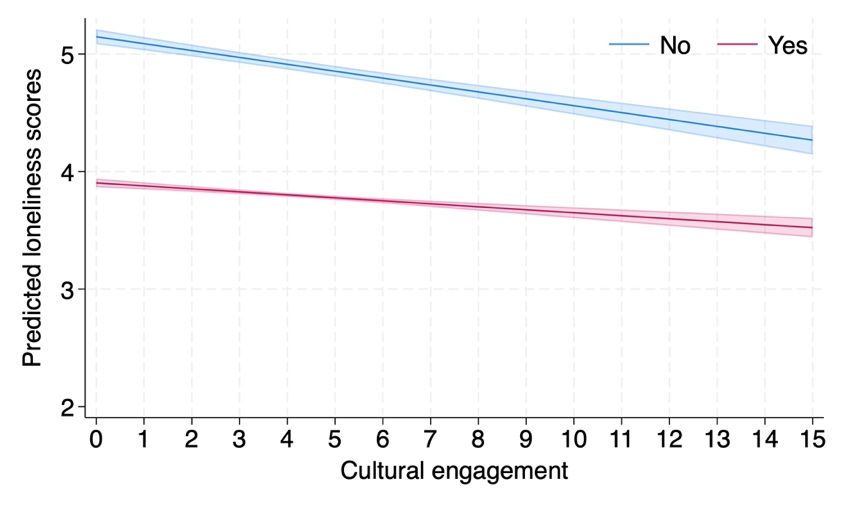

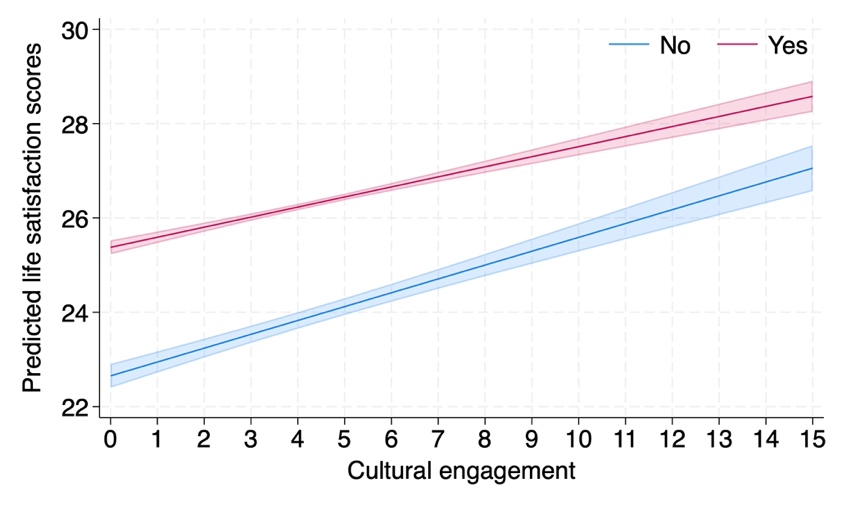

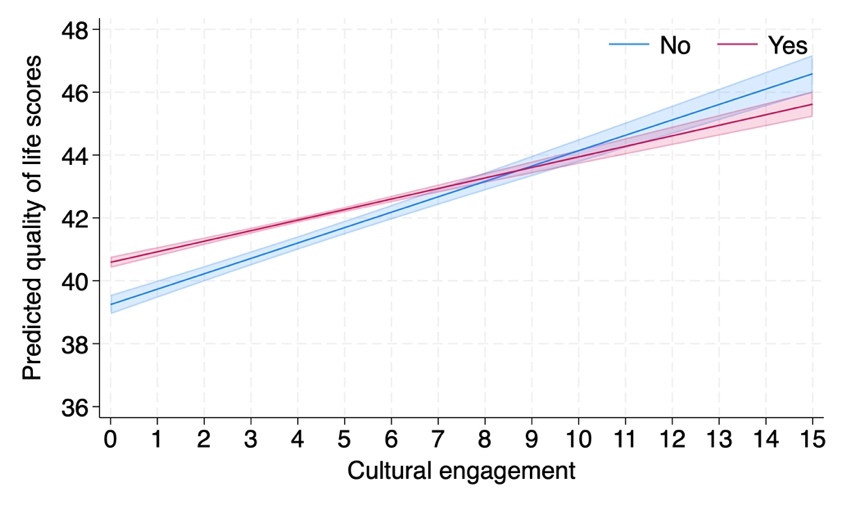

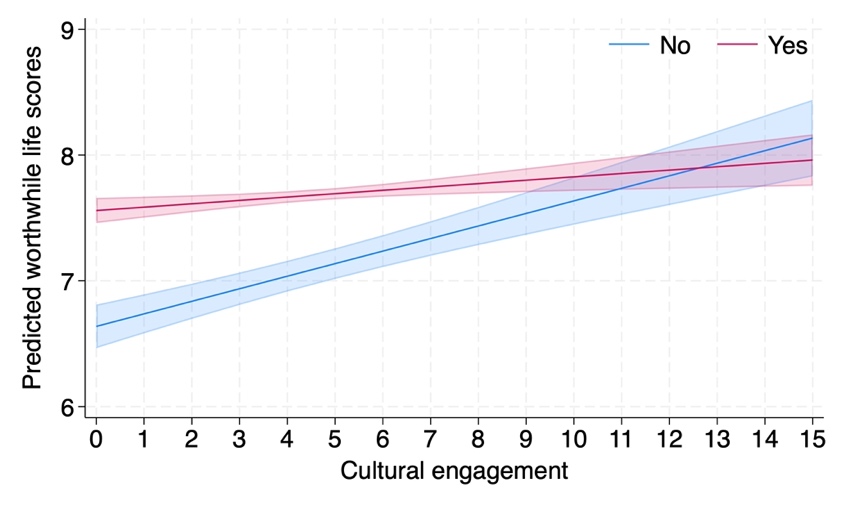


Alt Text: Scatterplot graphs showing living with partner status moderating the associations between cultural engagement with depressive symptoms, loneliness, life satisfaction, quality of life, and worthwhile life.

## **Supplementary Figure 5.** Interactions graphs for health status.


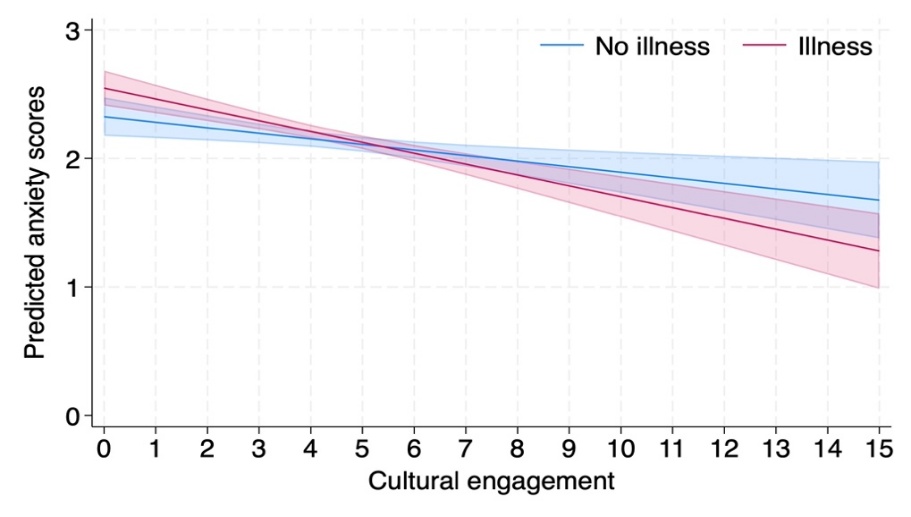

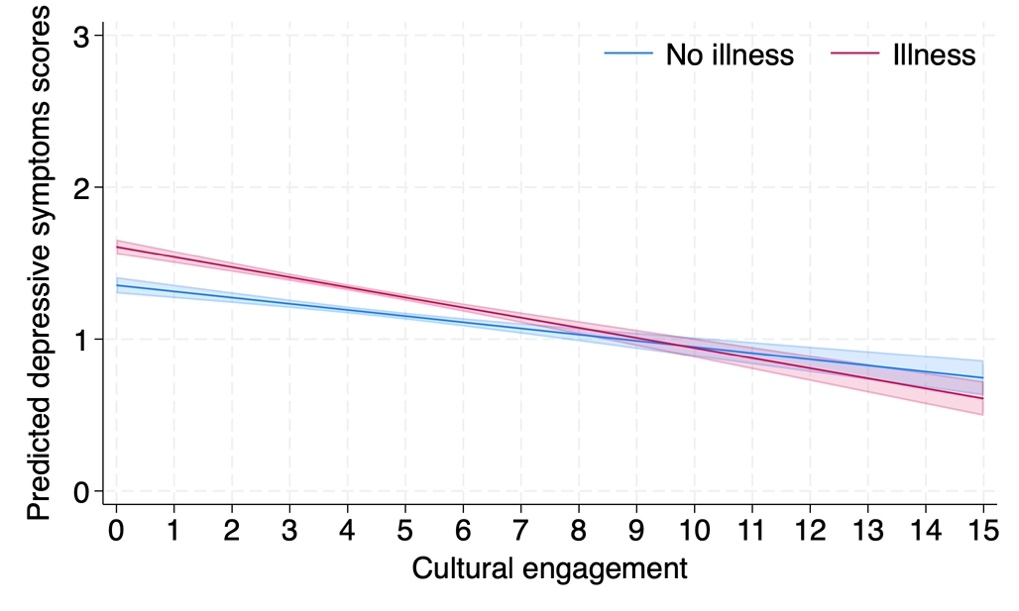

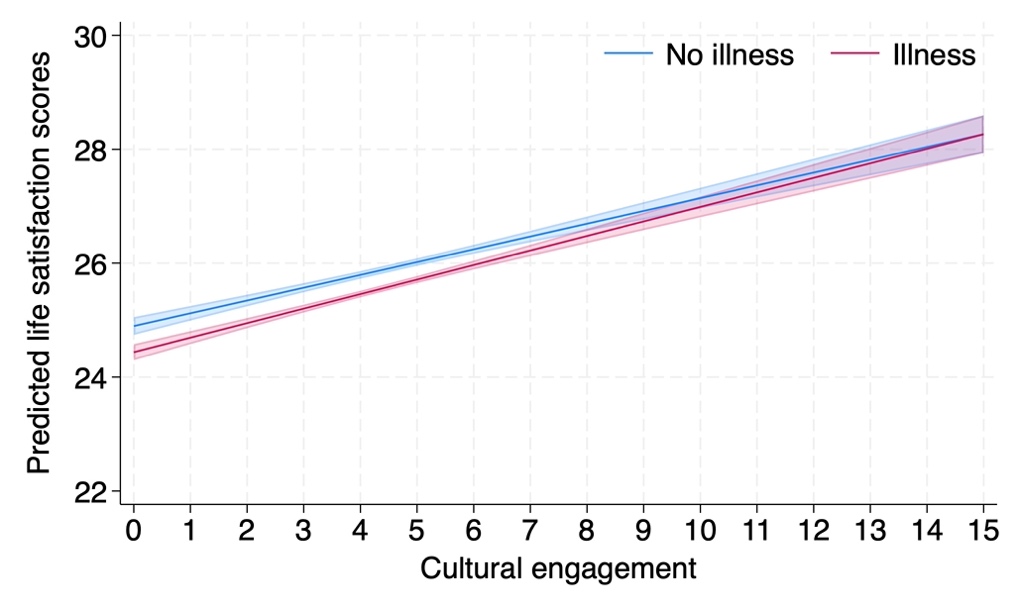

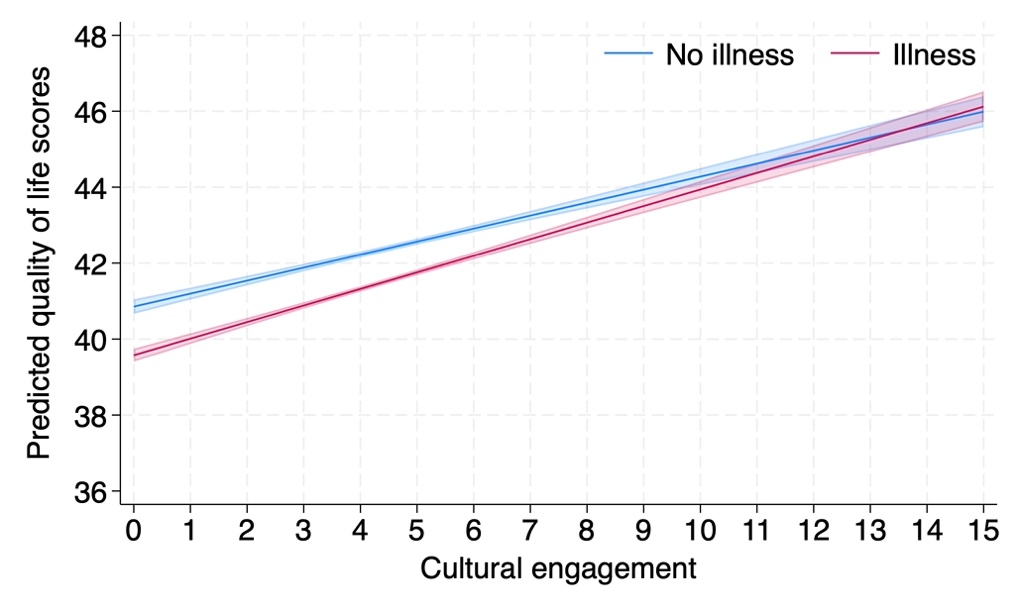


Sample 1 is n=10,428 and Sample 2 is n=6,932. These estimates are from fixed effects models and present within-person variations, showing the longitudinal associations between changes in cultural engagement and changes in the outcomes. Fully-adjusted models controlled for age, wealth, home ownership, employment status, and living with a partner.

Alt Text: Scatterplot graphs showing health status moderating the associations between cultural engagement with depressive symptoms, life satisfaction, quality of life, and anxiety.
